# Indigenous and faith healing for mental health in Ghana: An examination of the literature on reported beliefs, practices and use of alternative mental health care in Ghana

**DOI:** 10.4102/phcfm.v11i1.1941

**Published:** 2019-07-15

**Authors:** Lily Kpobi, Leslie Swartz

**Affiliations:** 1 Department of Psychology, Stellenbosch University, Stellenbosch, South Africa

**Keywords:** mental health, faith healing, indigenous, Ghana, beliefs, practices

## Abstract

**Background:**

For many people in African countries, various forms of health care are utilised for the treatment of illness. This pluralistic nature of health seeking includes the use of indigenous, faith and allopathic medicines for care.

**Aim:**

In this article, our aim was to gain insight into the existing knowledge on indigenous and faith healing in Ghana, with a particular focus on mental health care. We first examine the reported mental health beliefs and practices of Ghanaian alternative healers. Following this, we look at the use and purported preference for non-biomedical mental health care by patients.

**Methods:**

Relevant literature was examined to explore the beliefs, practices and use of non-biomedical mental health care systems in Ghana

**Results:**

Evidence for the use and preference for non-biomedical mental health care is largely anecdotal. Similarly, the mental health beliefs of alternative healers have been documented in various small-scale studies. However, such information is important if mental health services in Ghana are to be improved.

**Conclusion:**

Integration of the different healthcare systems must be built on knowledge of beliefs and methods. A clearer understanding of the work of non-biomedical healers is important if appropriate recommendations are to be made for collaboration between biomedical and non-biomedical systems in Ghana.

## Introduction

Similar to the case in other African countries, health care in Ghana is pluralistic, with people making use of indigenous, faith and allopathic healing systems for the treatment of illness.^1,2,3^ The indigenous systems often include components such as cultural norms and beliefs which have formed part of the everyday lives of people within communities.^4^ Biomedicine in Ghana was introduced during the colonial era, and brought with it western ideas of illness and healing.^2,5,6^ Consequently, indigenous Ghanaian notions and methods of health care were discouraged and considered ineffective. The colonial era also included the advent of Christianity in Ghana, through whose missions many traditional and cultural practices were prohibited as they were considered primitive and/or demonic.^7,8^ These prohibitions included the use and practice of indigenous methods of health care. To some extent, such notions of indigenous practices as primitive still exist in current discourse in Ghana, as seen in the tendency to refer to indigenous and faith healing systems as ‘alternative’ or ‘complementary’ to biomedicine. These systems also tend to be viewed as unchanging and static.

Kirmayer^9^ argues that the influence of increased globalisation has modified the association between what may be considered indigenous healing, and the underlying cultural beliefs on which they are thought to have been developed. A view of indigenous and traditional healing systems as rooted in fixed cultural practices is therefore problematic, as it reduces the heterogeneity of knowledge systems without reflecting growth and interaction.^10^ Although we recognise this challenge, for practical reasons (and to simplify engagement with the literature), we use the terms ‘indigenous healing’ and ‘traditional healing’ somewhat interchangeably to represent systems which have been developed within specific communities, and which represent culturally accepted forms of healthcare practice.

Furthermore, although indigenous healthcare practices are arguably predicated on traditional religious beliefs, for ease of presentation, the term ‘faith healing’ is used in reference to the practices of healers whose faith is drawn from non-indigenous religions. This is because religions like Pentecostal/Charismatic Christianity and Islam, as practised in Africa, are arguably syncretic in nature, and possess distinct and acknowledged external influences.

Various types of traditional and faith healers are recognised in Ghana.^11^ Although healers with specific specialisations exist (e.g. traditional birth attendants and traditional surgeons or bonesetters), we make reference to the categories of healers whose work includes (but is not necessarily limited to) mental health care. These categories include traditional herbalists, shrine devotees or diviners (often referred to as ‘fetish priests’ in Ghana), Pentecostal Christian faith healers and Muslim clerics or healers. These healers use different methods in their work, based largely on their training and orientation. The herbalists and diviners are widely viewed as cultural experts, and thus have elements of folk knowledge, cultural values and societal expectations of behaviour in their work.^12,13^

The Christian religion is not indigenous to Ghana; however, the churches that undertake faith healing typically belong to the neo-prophetic or charismatic tradition, which espouses a somewhat syncretic doctrine of Christianity. The neo-prophetic movement grew out of the Pentecostal movement of the mid-20th century, with a doctrine which stressed pneumatological manifestations of faith. Much emphasis was thus placed on the work of the Holy Spirit and its gifts of prophecy, speaking in tongues and miracles (including healing) as signs of good Christian living. Given that traditional African religion also emphasised concepts which manifested similarly (e.g. spirit possession or trances),^14^ there was much identification with the concepts that were taught by these churches. This similarity in style may be partly responsible for the fact that the charismatic movement grew in popularity in many African countries such as Ghana, particularly within the past four decades, though there are arguably other reasons for this popularity as well.^15^

In an examination of the growing phenomenon of charismatism in Ghana, Anderson^16^ observed similarities between the expression of neo-prophetic Christianity and what has been described as traditional African religion. The syncretic nature of many charismatic churches was shown in their emphasis on spiritual agents, confession, and the use of oils and holy water, which are common also in traditional African religious practice. An in-depth discussion of the syncretism of the charismatic movement is beyond the scope of this article, but more detailed discussion can be found in the works of Asamoah-Gyadu,^17^ Gifford^18^ and Omenyo, among others.^19^ Similar to Christianity, the nature of Islam in Ghana has also been argued to involve elements of indigenous Ghanaian ideas and practices.^20^ The work of healers who identify with these faiths are therefore influenced by both religious and cultural elements.^21^

This article is the first of two articles that examine indigenous and faith healing in alternative mental health care within the Ghanaian pluralistic context. In this article, we start by examining reported Ghanaian illness beliefs and the treatment methods that are used by indigenous and faith practitioners, with a focus on mental disorders. Next is an assessment of the literature on the use of and preference for alternative medicine by patients, and the reasons which have been posited as accounting for this preference. In the second article in this series, we present a brief history of the formalising process for traditional medical systems in Ghana, and conclude with an examination of collaborative efforts between biomedical systems and indigenous and faith healing systems in Ghana.

### Ethical considerations

This article followed all ethical standards for a research without direct contact with human or animal subjects.

## The mental health beliefs and practices of Ghanaian indigenous healers

The work of indigenous healthcare practitioners is predicated on their beliefs about the nature and cause of illness. For indigenous healers, the beliefs which inform their work are often rooted in cultural ideas and societal values which are shared by the communities that they serve. According to Omonzejele,^22^ in indigenous African systems, stability in an individual’s physical, mental, spiritual and social life is indicative of good health. The experience of instability in any one of these areas of the individual’s life is therefore likely to be experienced as an episode of illness. Similar to other African countries, in Ghana indigenous ideas of illness occurrence are often perceived when there is an imbalance in two or more components of a person’s life.^23,24,25^ The different components of a well-balanced life include physical and emotional well-being, social and communal harmony, as well as harmony with the gods and the spirits of the ancestors.^8,25^ A disruption of this harmony may result in physical or spiritual ailments, as well as in mental illness.^26^

Ghana has been described as a highly superstitious and religious country.^17,18,27^ As such, some early reports of Ghanaian beliefs about mental illness placed much emphasis on the belief in witchcraft and curses as explanations of mental illness (see, for instance, Field).^28,29^ Within such belief systems, mental illness is construed as resulting from the influence of spiritual entities whose purpose is to cause harm or to punish the individual for some wrongdoing. Such spiritual machinations would typically manifest in unexpected and disruptive behaviours.^30^

Despite the reported emphasis on spiritual explanations of mental disorders, there have been some studies which suggested that there was an awareness of other causal explanations. For instance, Quinn’s^31^ findings in a study of two urban and two rural communities in Ghana showed a higher acknowledgement of biomedical causes for mental disorders in three of the four communities, with less than 30% of respondents in each group endorsing spiritual elements as causes. Similarly, Kyei et al.^32^ reported a higher endorsement of social factors such as work stress and marital problems, accounting for mental illness.

However, the belief in spiritual causes for mental disorders cannot be ignored. A cursory look at the billboards and posters in many Ghanaian communities point to the large number of religious (in particular, neo-prophetic/charismatic Christian) centres which offer healing for various ailments including mental problems. These ‘prayer camps’ centre around a prophet who is believed to serve as a medium through which God heals people.^20,27^ Similarly, the presence of healers of other religious persuasions suggests that spiritual beliefs about mental disorders are a significant part of the lives of many Ghanaians. Therefore, the beliefs about illness include social as well as spiritual beliefs. In a review of mental health literature in Ghana, Read and Doku^33^ argued that Ghanaian beliefs about the nature and cause of mental disorders involved multiple, often fluid elements, unlike the perceived reliance solely on supernatural explanations that had previously been assumed.

There have been few studies exploring the specific treatment methods of indigenous and alternative healers in Ghana. In general, the work of indigenous healers has been described as involving herbal remedies, spiritual engagements, confession, as well as drawing on folk knowledge to restore social balance.^34,35^ Although some differentiation may exist in terms of the use of herbal versus spiritual methods, Hampshire and Owusu^36^ argue that this distinction is largely due to regulatory requirements, as many healers possess some knowledge of both systems but make use of specific methods based on the needs of each patient. However accurate or inaccurate this assertion may be, it seems to be limited to the work of herbalists and diviners. Faith healers have been reported to use more clearly spiritual methods. For instance, Osafo et al.^37^ examined the treatment regimens of Pentecostal pastors in Ghana for managing mental disorders, and reported a strong emphasis on prayer, fasting and the use of oils, candles and holy water, with no specific mention of herbal treatments. Similar methods were reported by Kpobi and Swartz.^38^ These methods are typically used in a prayer camp where patients stay while undergoing treatment.^27,39^ Similarly, Muslim healers in Ghana reportedly use specific prayers and verses from the Qur’an to treat illness.^40,41^

The differences in methods are also reflected in the way the different healers diagnose illnesses. In an earlier article, we discussed the ways in which different types of healers identify and classify different mental disorders.^42^ The authors identified differences between herbalists, diviners, Pentecostal pastors and Islamic healers with regard to classification of different types of biomedically classified mental disorders. Although psychotic disorders were clearly agreed on as indicative of mental problems, most of the healers did not consider common mental disorders as illness.^42^ These disorders were typically attributed to social circumstances. This suggests that the classification and diagnosis of different disorders is connected to the healing orientation of the healer which is often founded on cultural ideas of illness and wellness. In Ghanaian cultural belief systems, good health appears to encompass social, spiritual and physical balance and stability. Conversely, any instability or imbalance in one or more of these spheres of an individual’s life can be perceived as illness.

Ghanaian beliefs and methods with regard to mental illness, therefore, include physiological, social and spiritual explanations. These explanations are not always fixed; they are often fluid depending on the illness experience of the individual or their family. Understanding how illness is conceptualised can help to explain the type of help that is sought for health care. In the next section, we discuss the reported use of indigenous and faith health services by people in Ghana.

## The use of indigenous and faith healing by patients in Ghana

Various authors have examined why people in different African countries choose to use medical systems other than biomedical care.^43,44,45^ Although the literature is limited, the reasons for the choice of care in Ghana reflect similar trends as has been reported in other African countries. It has been argued that alternative systems of care are often the first point of call for people who view biomedicine as foreign.^46,47,48^ The ‘foreign’ nature of biomedicine was perceived not to reflect indigenous beliefs about the causes of illness.

Beyond similarities in illness beliefs, biomedical care was also argued to be less easily accessible for a significant number of people, thus leading people to opt for care from indigenous healers.^14,49^ Estimates of the human resources for biomedical care suggest that there is approximately one psychiatrist for every 1 million people in Ghana, with similar ratios of psychologists and social workers being reported, although there were slightly more mental health nurses working at various levels.^50^ Furthermore, a large proportion of these professionals work in the relatively few urban sectors of the country, leaving a significant portion of the rural population with limited access to formal biomedical care. Given these estimated human resource limitations, Ofori-Atta et al.^51^ have projected that only 2% of Ghanaians requiring mental health care had access to the needed help. Thus, a large proportion of the Ghanaian populace was thought to rely on non-biomedical health care, as formal biomedical care was simply not available.

Even for those who had access to biomedical facilities, another factor which has been suggested to account for the choice to use indigenous and faith healing for mental illness is the perceived high cost of biomedical services.^52,53^ Some authors have suggested that the flexibility and the modes of payment accepted by indigenous healers were preferable to patients, as these enabled a type of barter system to be used,^12^ even when the ultimate cost was higher than the biomedical cost. Patients were able to pay for services by offering to exchange them for poultry or other animals, pieces of fabric, or availing themselves to help with chores and other tasks that the healer may require.^13,54,55^

The general perception in early literature has therefore been that people chose to seek help from indigenous healers first because the biomedical services did not align with their beliefs.^1^ However, in a more recent study of patients presenting to four facilities which provided mental health services in Kumasi, Appiah-Poku et al.^56^ reported that fewer than 20% of patients had sought help from alternative healers for the first episode of illness. The majority of patients sought help from biomedical facilities despite the assumed supernatural illness beliefs. Likewise, in other studies Read^57,58^ observed that many patients reportedly sought treatment from indigenous or faith healers only when the biomedical methods did not meet their expectations or because of the perceived limited efficacy of psychotropic medications. Read^57^ therefore argued that the pathways for mental health care were often driven not so much by illness beliefs about cause, but rather by expected outcomes and the desire for permanent solutions to their health problems.

## Conclusion

Non-biomedical mental health care is obviously very popular in Africa, including in Ghana. In this article, we sought to provide some insights into the different dimensions of indigenous and faith healing in the Ghanaian context from published literature. Knowledge of these components of alternative care is important in light of the calls for improved care and the narrowing of the mental health treatment gap in Ghana. In particular, efforts at collaboration between the different systems must be built on accurate understandings of the perspectives of key stakeholders. As mentioned above, this article is the first part of a review of the literature on indigenous and faith healing in Ghana. In the next article, we will examine the ways in which indigenous health services in Ghana have been organised and the role that such organisations play in collaborative efforts which have been attempted in Ghana.

This article was not a systematic review of the literature on indigenous and faith healing in Ghana. Instead, our aim was to gain insight into the existing knowledge on alternative healing for mental health in Ghana. We therefore do recognise that some nuance may have been overlooked, such as the absence of the rigour that a systematic review lends to analysing literature, but we anticipate that this discussion will contribute meaningfully to the conversation on mental health care (in all its forms) in Ghana.

## References

[1] Aninyam C Traditional medical practice in contemporary Ghana: A dying or growing ‘Profession’? Can J Afr Stud. 1987;21(3):315–336.

[2] Busia K Medical provision in Africa: Past and present. Phytother Res. 2005;19:919–923. 10.1002/ptr.1775 16317644

[3] Evans-Anfom E Traditional medicine in Ghana: Practice, problems and prospects. Accra: Academy of Arts and Sciences; 1986.

[4] Gyasi RM, Mensah CM, Adjei PO, Agyemang S Public perceptions of the role of traditional medicine in the health care delivery system in Ghana. Glob J Health Sci. 2011;3(2):40–49. 10.5539/gjhs.v3n2p40

[5] Jahoda IG Traditional healers and other institutions concerned with mental illness in Ghana. Int J Psychiatry. 1961;7:245–268. 10.1177/002076406100700401

[6] Twumasi PA Medical systems in Ghana: A study in medical sociology. Tema: Ghana Publishing; 1975.

[7] Asamoah-Gyadu JK Therapeutic strategies in African religions: Health, herbal medicines and indigenous Christian spirituality. Stud World Christianity. 2014;20(1):70–90. 10.3366/swc.2014.0072

[8] Fink HE Religion, disease and healing in Ghana: A case study of traditional Dormaa medicine. München: Tricker Wissenschaft; 1990.

[9] Kirmayer LJ The cultural diversity of healing: Meaning, metaphor and mechanism. Br Med Bull. 2004;69:33–48. 10.1093/bmb/ldh006 15226195

[10] Orr DMR, Bindi S Medical pluralism and global mental health In: White R, Jain S, Orr D, Read U, editors The Palgrave handbook of sociocultural perspectives on global mental health. London: Palgrave Macmillan, 2017; p. 307–328.

[11] Addy ME Putting science into the art of healing with herbs. Accra: Ghana Universities Press; 2003.

[12] Konadu K Indigenous medicine and knowledge in African society. New York: Routledge; 2007.

[13] Tabi MM, Powell M, Hodnicki D Use of traditional healers and modern medicine in Ghana. Int Nurs Rev. 2006;53:52–58. 10.1111/j.1466-7657.2006.00444.x 16430761

[14] Appiah-Kubi K Man cures, God heals: Religion and medical practice among the Akan of Ghana. New York: Friendship Press; 1981.

[15] Zalanga S Civil society in Africa: Interrogating the role of Pentecostal Christianity in Africa’s democratization and development processes In: Kieh G Jr., editor Contemporary issues in African society. Cham: Palgrave Macmillan, 2018; p. 47–81.

[16] Anderson A An introduction to Pentecostalism. Cambridge: Cambridge University Press; 2004.

[17] Asamoah-Gyadu JK Contemporary Pentecostal Christianity: Interpretations from an African Context. Oxford: Regnum Books; 2013.

[18] Gifford P Ghana’s new Christianity: Pentecostalism in a globalizing African economy. Indianapolis, IN: Indiana University Press; 2004.

[19] Omenyo CN Pentecost outside Pentecostalism: A study of the development of charismatic renewal in the mainline churches in Ghana. Zoetermeer: Boekencentrum; 2006.

[20] Read UM Madness and miracles: Hoping for healing in rural Ghana In: Littlewood R, Lynch R, editors Cosmos, gods and madmen: Frameworks in the anthropologies of medicine. Oxford: Berghahn Books, 2016; p. 45–66.

[21] Mullings L Therapy, ideology and social change: Mental healing in urban Ghana. Los Angeles, CA: University of California Press; 1984.

[22] Omonzejele PF African concepts of health, disease, and treatment: An ethical inquiry. Explore. 2008;4(2):120–123. 10.1016/j.explore.2007.12.001 18316055

[23] Opare-Henaku A, Utsey SO Culturally prescribed beliefs about mental illness among the Akan of Ghana. Transcult Psychiatry. 2017;54(4):502–522. 10.1177/1363461517708120 28612682

[24] Pobee JS Health, healing and religion: An African view. Int Rev Missions. 2001:356–357. 10.1111/j.1758-6631.2001.tb00260.x

[25] White P The concept of diseases and health care in African traditional religion in Ghana. HTS Teologiese Studies/Theol Stud. 2015;71(3): 2762 10.4102/hts.v71i3.2762

[26] Akpomuvie BO The perception of illness in traditional Africa and the development of traditional medical practice. Int J Nurs. 2014;1(1):51–59.

[27] Arias D, Taylor L, Ofori-Atta A, Bradley EH Prayer camps and biomedical care in Ghana: Is collaboration in mental health care possible? PLoS One. 2016;11(9):e0162305 10.1371/journal.pone.0162305 27618551 PMC5019394

[28] Field MJ Witchcraft as a primitive interpretation of mental disorder. J Ment Sci. 1955;101:826–833. 10.1192/bjp.101.425.826 13271974

[29] Field MJ Search for security: An ethno-psychiatric study of rural Ghana. London: Faber & Faber; 1960.

[30] Ventevogel P, Jordans M, Reis R, De Jong J Madness or sadness? Local concepts of mental illness in four conflict-affected African communities. Confl Health. 2013;18:3 10.1186/1752-1505-7-3 PMC3605182 23418727

[31] Quinn N Beliefs and community responses to mental illness in Ghana: The experiences of family carers. Int J Soc Psychiatry. 2007;53(2):175–188. 10.1177/0020764006074527 17472090

[32] Kyei JJ, Dueck A, Indart MJ, Nyarko NY Supernatural belief systems, mental health and perceptions of mental disorders in Ghana. Int J Cult Ment Health. 2014;7(2):137–151. https://doi.org10.1080/17542863.2012.734838

[33] Read UM, Doku VCK Mental health research in Ghana: A literature review. Ghana Med J. 2012;46(2):29–38. 23661815 PMC3645145

[34] Aniah P The contribution of indigenous health care providers to health care delivery in rural Ghana: An exploratory study of Bongo District. Sci J Public Health. (Special Issue: Health Behaviour and Public Health). 2015;3(1–1):20–28. 10.11648/j.sjph.s.2015030101.14

[35] Kpobi L, Swartz L ‘That is how the real mad people behave’: Beliefs about and treatment of mental disorders by traditional medicine men in Accra, Ghana. Int J Soc Psychiatry. 2018;64(4):309–316. 10.1177/0020764018763705 29529921

[36] Hampshire KR, Owusu SA Grandfathers, Google and dreams: Medical pluralism, globalization and new healing encounters in Ghana. Med Anthropol. 2013;32(3):247–265. 10.1080/01459740.2012.692740 23557008

[37] Osafo J, Agyapong M, Asamoah MK Exploring the nature of treatment regimen for mentally ill persons by neo-prophetic ministers in Ghana. Int J Cult Ment Health. 2015;8(3):325–339. 10.1080/17542863.2014.973428

[38] Kpobi LNA, Swartz L ‘The threads in his mind have torn’: Conceptualization and treatment of mental disorders by neo-prophetic Christian healers in Accra, Ghana. Int J Ment Health Syst. 2018;12:40 10.1186/s13033-018-0222-2 30061921 PMC6056911

[39] Edwards J Ghana’s mental health patients confined to prayer camps. Lancet. 2014;383:15–16. 10.1016/S0140-6736(13)62717-8 24400332

[40] Adu-Gyamfi S Islamic traditional healing amongst the people of Sampa in Ghana: An empirical study. Online Int J Arts Human. 2014;3(7):105–114.

[41] Kpobi LNA, Swartz L Muslim traditional healers in Accra, Ghana: Beliefs about and treatment of mental disorders. J Relig Health. 2018;58(3):833–846. 10.1007/s10943-018-0668-1 29992474

[42] Kpobi L, Swartz L Explanatory models of mental disorders among traditional and faith healers in Ghana. Int J Cult Ment Health. 2018;11(4):605–615. 10.1080/17542863.2018.1468473

[43] Burns JK, Tomita A Traditional and religious healers in the pathway to care for people with mental disorders in Africa: A systematic review and meta-analysis. Soc Psychiatry Psychiatr Epidemiol. 2014;50(6):867–877. 10.1007/s00127-014-0989-7 25515608 PMC4442066

[44] Kajawu L, Chingarande SD, Jack H, Ward C, Taylor T What do African traditional medical practitioners do in the treatment of mental disorders in Zimbabwe? Int J Cult Ment Health. 2016;9(1):44–55. 10.1080/17542863.2015.1106568

[45] Mbwayo AW, Ndetei DM, Mutiso V, Khasakhala LI Traditional healers and provision of mental health services in cosmopolitan informal settlements in Nairobi, Kenya. Afr J Psychiatry. 2013;16:134–140. 10.4314/ajpsy.v16i2.17 23595533

[46] Ae-Ngibise K, Cooper S, Adiibokah E, Akpalu B, Lund C, Doku V ‘Whether you like it or not people with mental problems are going to go to them’: A qualitative exploration into the widespread use of traditional and faith healers in the provision of mental health care in Ghana. Int Rev Psychiatry. 2010;22:558–567. https://doi.org/0.3109/09540261.2010.536149 21226644 10.3109/09540261.2010.536149

[47] Ewusi-Mensah I Postcolonial psychiatric care in Ghana. Psychiatrist. 2001;25:229–231.

[48] Fiasorgbor DA, Aniah SA Perceptions and beliefs about mental illness (schizophrenia) among adults in Zaare Community. Dev Country Stud. 2015;5(9):151–158.

[49] Biritwum RB, Jackson HE Health care accessibility and utilization in sub-Saharan Africa: Experiences from Ghana In: Kalipeni E, Thiuri P, editors Issues and perspectives on health care in contemporary sub-Saharan Africa. Lampeter, PA: Edwin Mellen Press, 1997; p. 361–374.

[50] Roberts M, Morgan C, Asare JB An overview of Ghana’s mental health system: Results from an assessment using the World Health Organization’s Assessment Instrument for Mental Health Systems (WHO-AIMS). Int J Ment Health Syst. 2014;8:16 10.1186/1752-4458-8-16 24817908 PMC4016652

[51] Ofori-Atta A, Read UM, Lund C A situation analysis of mental health services and legislation in Ghana: Challenges for transformation. Afr J Psychiatry. 2010;13:99–108. 10.4314/ajpsy.v13i2.54353 20473470

[52] Oppong S, Kretchy IA, Imbeah EP, Afrane BA Managing mental illness in Ghana: The state of commonly prescribed psychotropic medicines. Int J Mental Health Syst. 2016;10(1):28 10.1186/s13033-016-0061-y PMC4820990 27051464

[53] Read UM, Adiibokah E, Nyame S Local suffering and the global discourse of mental health and human rights: An ethnographic study of responses to mental illness in rural Ghana. Glob. Health. 2009;5(1):13 10.1186/1744-8603-5-13 PMC2770454 19825191

[54] Bierlich BM Injections and the fear of death: An essay on the limits of biomedicine among the Dagomba of northern Ghana. Soc Sci Med. 2000;50:703–713. 10.1016/S0277-9536(99)00322-6 10658850

[55] Bierlich BM The problem of money: African agency and western medicine in northern Ghana. New York: Berghahn Books; 2007.

[56] Appiah-Poku J, Laugharne R, Mensah E, Osei Y, Burns T Previous help sought by patients presenting to mental health services in Kumasi, Ghana. Soc Psychiatry Psychiatr Epidemiol. 2004;39:208–211. 10.1007/s00127-004-0725-9 14999453

[57] Read UM ‘I want the one that will heal me completely so it won’t come back again’: The limits of antipsychotic medication in rural Ghana. Transcult Psychiatry. 2012;49(3–4):438–460. 10.1177/1363461512447070 22722982

[58] Read UM ‘Doctor sickness’ or ‘Pastor sickness’? Contested domains of healing power in the treatment of mental illness in Kintampo, Ghana In: Basu H, Littlewood R, Steinforth AS, editors Spirit and mind: Mental health at the intersection of religion and psychiatry. Munster, Germany: LIT verlag, 2017; p. 167–188.

